# The management of muscle-invasive bladder Cancer is still a significant challenge in the clinical practice

**DOI:** 10.1590/S1677-5538.IBJU.2021.0329.1

**Published:** 2021-12-21

**Authors:** Arie Carneiro

**Affiliations:** 1 Hospital Israelita Albert Einstein Departamento de Urologia São Paulo SP Brasil Departamento de Urologia, Hospital Israelita Albert Einstein, São Paulo, SP, Brasil

## COMMENT

The authors should be congratulated on reporting their findings in a large radical cystectomy cohort for muscle-invasive (MIBC) and metastatic bladder cancer (1). The management of MIBC is still a significant challenge in the clinical practice. Although treatment options have a high rate of oncological success, they are related to a negative impact on patients’ quality of life. Thus, correct staging is mandatory to select the appropriate treatment for each patient. The presence or absence of lymph node involvement directly correlates with the patient's oncological prognosis and can also impact the treatment choice. The local treatment is the key to success in patients with localized disease, with a preference for cystectomy plus lymph node dissection associated with neoadjuvant chemotherapy when indicated. For patients with lymph node involvement, chemotherapy is the mainstay of treatment, and local treatment is evaluated in a second moment for selected cases. The authors analyzed preoperative contrast-enhanced scans of patients undergoing radical cystectomy from 2004 to 2019 in a tertiary high-volume center. The local tumor stage and lymph node (LN) characteristics such as size, morphology (MLN) and number of loco-regional LN (NLN) were investigated and correlated to lymph node ratio (LNR) and survival. The main finding was that the presence of lymph nodes larger than 15 mm was the only statistically significant factor related to neoplastic lymph node involvement. The overall accuracy for local tumor stage and LN metastasis detection was 62% and 72%, respectively. This is the first report to demonstrate that the number of loco-regional lymph nodes in a contrast-enhanced (CT) scan is correlated with decreased CSS and OS (p=0.001: p=0.002). Aaccording to the AUA guidelines, lymph nodes above 8 mm should be considered suspicious, however with poor accuracy, with sensitivity between 48 and 87%, besides there may be larger benign lymph nodes as well as lymph nodes smaller than 8mm with microscopic neoplastic infiltration (2, 3). Importantly, most patients were treated in an era in which neoadjuvant therapy was not routinely performed. In current series, the rate of positive lymph node is lower, and, probably, this may be one of the reasons for the oncological benefit of neoadjuvant therapy, adding the benefit of systemic treatment in patients with supposedly localized disease. Another important factor to be considered in this scenario is the use of PET scan. The value of PET in MIBC is mentioned in the literature, but its practical application is still controversial, mainly due to how different groups conduct the exam (whether they do it or not after diuretic imaging), which is of huge importance for good results, among other aspects. Some studies that compared PET with CT showed the superiority of PET with an accuracy of 84% vs 78%, respectively, (p=0.039) (4, 5). In conclusion, I believe that this paper findings highlights the CT scan's accuracy and report a new characteristic to be observed when staging MIBC. In additional, they report the importance of the size of the lymph node and the number of pelvic lymph nodes in the oncologic outcome. Despite these promising advances, this issue still needs to be addressed in future studies.

## References

[1] Eismann L, Rodler S, Tamalunas A, Schulz G, Jokisch F, Volz Y, Pfitzinger P, Schlenker B, Stief C, Solyanik O, Buchner A, Grimm T. Computed-tomography based scoring system predicts outcome for clinical lymph node-positive patients undergoing radical cystectomy. Int Braz J Urol. 2022;48:89-98. 10.1590/S1677-5538.IBJU.2021.0329 PMC8691251 34528776

[2] Barentsz JO, Engelbrecht MR, Witjes JA, de la Rosette JJ, van der Graaf M. MR imaging of the male pelvis. Eur Radiol. 1999;9:1722-36. 10.1007/s003300050916 10602944

[3] Dorfman RE, Alpern MB, Gross BH, Sandler MA. Upper abdominal lymph nodes: criteria for normal size determined with CT. Radiology. 1991;180:319-22. 10.1148/radiology.180.2.2068292 2068292

[4] Girard A, Rouanne M, Taconet S, Radulescu C, Neuzillet Y, Girma A, et al. Integrated analysis of 18F-FDG PET/CT improves preoperative lymph node staging for patients with invasive bladder cancer. Eur Radiol. 2019;29:4286-93. 10.1007/s00330-018-5959-0 30666449

[5] Pichler R, De Zordo T, Fritz J, Kroiss A, Aigner F, Heidegger I, et al. Pelvic Lymph Node Staging by Combined 18F-FDG-PET/CT Imaging in Bladder Cancer Prior to Radical Cystectomy. Clin Genitourin Cancer. 2017;15:e387-95. 10.1016/j.clgc.2016.08.009 27601364

